# Promoting sustainable development goals: Role of higher education institutions in climate and disaster management in Zimbabwe

**DOI:** 10.4102/jamba.v14i1.1206

**Published:** 2022-06-29

**Authors:** Munyaradzi A. Dzvimbo, Tinashe M. Mashizha, Kelvin Zhanda, Albert Mawonde

**Affiliations:** 1 Department of Development Studies, University of South Africa, Pretoria, South Africa; 2 Plant and AgriBiosciences Research Centre, Ryan Institute, National University of Ireland, Galway, Ireland; 3 Department of Architecture and Real Estate, University of Zimbabwe, Harare, Zimbabwe; 4 College of Agriculture and Environmental Sciences, University of South Africa, Pretoria, South Africa

**Keywords:** climate, disaster, higher education, management, sustainable development

## Abstract

This article seeks to explore the role of higher education institutions (HEIs) in climate change adaptation and disaster risk management (DRM). The study is based on the qualitative desk review, thematic and document analysis and uses the theory of change to facilitate a road map for HEIs in strengthening professional human development, policy consistency in dealing with climate-induced natural disasters (CINDs) research and policies. Academic journals covering the role of HEIs in climate and disaster management in Zimbabwe were selected on google scholar. Reviewed documents include universities’ curriculum documents, government disaster policy documents and other related disaster management policy protocols. The article’s findings reflect that HEIs in Zimbabwe have been engaged in climate change education and DRM as the country and the region are prone to threats posed by extreme weather events in the form of tropical cyclones and extreme droughts. The article concludes that HEIs core mission that includes public engagement to advance achieving sustainable development goals in Zimbabwe is growing at a steady pace to find ways to avert the impact of climate change and put strategies in place to respond to disaster to minimise social, environmental and economic losses posed by disasters. Bindura University of Science Education (BUSE) is enhancing people’s resilience in Chadereka village in Muzarabani through disaster preparedness training. National University of Science Technology (NUST) is also training communities on disaster concepts, disaster prevention, mitigation, preparedness and response.

## Introduction

Education and academia are springboards for sustainable development (SD) worldwide (Pappas 2012). It is beyond doubt and indisputable that higher education and training are fundamental to the preferment of Sustainable Development Goals (SDGs) related to disaster management and climate change (King 2011; Kupika, Mbereko & Chinokwetu 2020; Leal Filho et al. 2018; Mawonde & Togo 2019; UNESCO 2014). The importance of education is evident because it is a fundamental human right that is provided in the Bill of Rights for different countries across the world (Lee 2020). Globally, SDG4 envisions that by 2030 there will be all-encompassing and equitable quality education to support lifelong learning prospects for all (Kwauk 2020; Mashizha & Dzvimbo 2018; Mawonde & Togo 2019). The United Nations Department of Economic and Social Affairs (UN 2016) highlighted the need for partnerships among governments, citizenry and the private sector to accomplish available development programme (Jomo et al. 2016). As such, higher education institutions (HEIs) teaching, learning, research, innovation and community engagement need to be considered a driver for implementing SDGs to reduce the impact of climate-induced natural disasters (CINDs).

The strategic importance and relevance of academia in achieving SDGs has been recently repeated in various research by scholars such as Bhowmik, Selim and Huq (2017), Albareda-Tiana et al. (2020) and Kupika et al. (2020). The World Commission on Environment and Development (WCED) applauded higher education institutions for their critical role and involvement in political transformation, public awareness and the capability to bring the globe into a sustainable path (WCED 1987). The European University Association (EUA), which represents over 800 universities in European countries, regards universities as strong institutions accountable to society and capable of promoting SD (Brennan, King & Lebeau 2004). According to their efforts in promoting SDG achievement, the recent ranking of universities is driving the HEIs to achieve development (De la Poza et al. 2021).

The African Union Agenda 2063 aspires to promote SD and raise awareness on climate and disaster management through education (Africa Union Commission 2015). The Sendai Framework for Disaster Risk Reduction (SFDRR) 2015–2030 is crucial for climate disasters. The framework advocates for reducing global disaster mortality, direct economic loss concerning gross domestic product (GDP), disaster damage related to critical infrastructure and disruption of basic services and an increase in the number of countries with national and local disaster risk reduction strategies (Pearson & Pelling 2015). According to UNISDR Hyogo Framework for Action (2007), the paramount obligation of states is to put in place disaster risk management (DRM) strategies and frameworks at various levels to avert risks in communities posed by various natural disasters such as cyclones, droughts, tsunamis, earthquakes and volcanoes.

The Hyogo Framework of Action (HFA) of 2005–2015 focused on building resilience to disasters in nations and communities by 2015 through education and knowledge to enhance resilience (UNISDR Hyogo Framework for Action 2007). The framework provides a befitting conceptual underpinning for this study. These frameworks challenge the role of HEIs to the position of executing disaster management for SD through knowledge management, innovation, education and training, research, information exchange, dissemination and public awareness, national institutional frameworks and community participation (Tanesab 2020). The fundamental goal of the HFA is to challenge HEIs to consistently play a pivotal role in promoting SDG through the implementation of climate and disaster management curriculum, research projects and simulations.

Sustainable development is a critical pillar of socio-economic and environmental development in societies regarding DRM and climate change awareness and education (Mensah 2019). However, climate-induced disasters are threatening and derailing the efforts put by poor governments to respond accordingly to these challenges through proper sustainable strategies in Zimbabwe and other countries in the global South (GS). Disasters are environmental concerns that require special attention by higher education learning institutions, and their roles will go a long way in addressing the current institutional chasms in climate DRM. Disaster risk reduction and resilience are reflected in 25 targets and 10 of the 17 SDGs particular goals (UNISDR 2015).

This article seeks to parade and explain the absence of understanding of how higher education learning institutions can be used to deal with the continuing challenges of CINDs in Zimbabwe. Through higher education and learning avenues, especially research and development, communities can be empowered with knowledge that they can ultimately use to transform their lives and promote their well-being, especially in the light of changing climate and disasters (King 2011). Higher education and learning are broad and diverse and can be packaged in different forms by nature to suit climate change induced disaster management and resilience studies. This study focuses on environmental education and training (EET), a critical component of higher education and learning considering that climate, disasters and environmental issues are intrinsic components of SD.

While the *Civil Protection Act* (1989) in Zimbabwe bestows responsibility to every citizen to assist the reduction of disasters (Simba 2018), the HEIs have a critical and unique role to play in climate and disaster management in Zimbabwe (Fitzgerald et al. 2017).

## Literature review

### Tertiary institutions in Zimbabwe

Higher learning and education institutions in Zimbabwe are under the Ministry of Higher and Tertiary Education, Science and Technology jurisdiction. According to the Zimbabwe Council for Higher Education (ZIMCHE) Act of (2006), there are 24 registered universities in Zimbabwe, and among them, 14 are public institutions and 10 are private (Garwe & Thondhlana 2019). The ZIMCHE oversees these education entities for quality promotion and accreditation, among other services. Given that there are many HEIs in Zimbabwe, there is a great need to focus on their roles in as far as climate change, disaster management and ultimately how they are promoting the achievement of SDGs in Zimbabwe.

Higher education institutions have a crucial role in providing knowledge, evidence-based solutions and innovation to facilitate SDGs attainment through extensive research activities and capabilities (Albareda-Tiana et al. 2020; Bhowmik et al. 2017; Leal Filho et al. 2018). Co-responsibility, collaborations and joint programmes with higher learning institutions would be vital in realising SD (Trencher et al. 2014) through climate and DRM. For example, in 2021, Bindura University of Science Education (BUSE) in Mashonaland Central Zimbabwe collaborated with the Ministry of Environment in Climate Change, Collaboration Pact to tackle the climate change phenomenon. The collaboration will see BUSE leading the role of conducting researches on climate change. A non-governmental organisation such as the World Wide Fund for Nature (WWF) partners with various universities in climate change and DRM strategies by providing disaster risk expertise and funding. Various stakeholders in Zimbabwe recognise the need for education to institute sustainable economic transformation, as shown by student scholarships such as the Joshua Nkomo and Econet Wireless Zimbabwe scholarships supporting studies in science, among other areas.

### Climate change induced disasters and the role of universities

Climate and disasters are intertwined through climate aberrations and sudden weather changes that vary over time. Climate variations and changes manifest or exhibit themselves in the form of extreme weather events such as droughts and floods (CINDs). Moreover, in most cases, these have the potential for loss of human lives and destruction of infrastructure (Diakakis et al. 2020). It is also vital to observe that climate variations and risky weather occasions have regularly occurred, resulting in property and livelihood losses (Matamanda, Dzvimbo & Kadebu 2017; UNESCO 2014; UNISDR 2009). The United Nations classifies disasters resulting from climatic variability and other climatic and meteorological causes as hydro-meteorological disasters (floods, landslides, mudflows, avalanches, tidal waves, windstorms, including typhoons, cyclones, hurricanes, storms, winter storms, tropical storms and tornadoes, droughts, extreme temperatures and complex disasters associated with drought) as distinct from geological disasters (earthquakes, volcanic eruptions and tsunamis) (Cui 2016; UNESCO 2014). The hydro-meteorological disasters resulting from climate variability and other climatic and meteorological causes are commonly referred to as ‘climate disasters’ in disaster studies. Climate disasters have always been a recurring theme in human history and are rising. As these natural disasters are rising, universities are challenged by various international calls to respond with innovative strategies to tackle and adapt to these natural disasters to save lives, infrastructure, economic growth, resilience and human safety. One such call is the inception of the SDGs in 2015, which challenged universities and HEIs to embrace and implement ways to use each goal to attain sustainability, peace, growth, equity and improved standards of living (Mawonde & Togo 2019). The SDG 4 (quality education), SDG 11 (sustainable cities and communities), SDG 13 (climate action) and SDG 17 (partnership for the goals) are some goals universities or HEIs can use to tackle natural disasters and climate-induced extreme weather events. Universities are known for innovation, knowledge production, academic prowess and they have the trust of the society, thus they are better placed to influence communities to adapt to DRM strategies and climate change adaption methods (Mawonde & Togo 2019).

### Sustainable development and climate disasters

Sustainable development is a process that requires us to view our lives as elements of a larger entity and our actions have a cumulative influence in building sustainable growth and systems or further degrading ecosystems and natural flow of resources to the detriment of humanity (Blewitt 2012). Sustainable development is about protecting and conserving the natural environment and promoting social equity and a degree of economic equality within and between nations (Chigundu 2018; Cui 2016). This can be conceptualised as a process of convergence, so the question of spatial scale is necessary in any serious thinking and action designed to make our world a better place. Sustainable development goals are targets and goals set to address various problems befalling all countries globally, including peculiar challenges faced by the GS by 2030 (Messerli et al. 2019). Australia and sub-Saharan Africa are examples of the GS affected by CINDs (Crompton & McAneney 2008; Graham 2020). They are hinged upon the idea that the future should be a better, healthier place than the present without severe natural disasters. Neither modern nor postmodern SD strategies requires an understanding of the natural world and the human social world as being not so much ‘connected’ as the same but compartmentalised such that there is a need to harness SD through HEIs to achieve meaningful ways to avert global sustainability challenges among them CINDs (Chirisa & Dumba 2012; Nhede 2013).

Benson (2016) explicates the formidable impacts of disasters on SD thus:

Disasters undermine sustainable development. They result in loss of life and cause injury, sometimes with life-changing consequences. Furthermore, they destroy homes, schools, health clinics, hospitals, utilities, roads, markets, and other social and economic infrastructure and damage the natural environment. These direct, physical losses have further indirect consequences, disrupting livelihoods, education, access to health care, and so forth, together with leading to adverse secondary impacts on social and economic aggregates such as GDP, the balance of payments, and budget deficits. (p. 2)

Against this background, the United Nations Resolution on SD calls for building resilience and disaster risk reduction in SD and poverty reduction (Aitsi-Selmi et al. 2015). Sustainable development stresses risk reduction initiatives and development is not sustainable if it does not consider DRM. So, to achieve SD towards harnessing CINDs, universities assumed the role to expand their core mission of teaching and learning, research, community engagement and knowledge creation to include innovation and creativity to respond to various challenges confronting humanity (Togo & Gandidzanwa 2021).

## Integrated disaster risk management framework

The possibility of climate and natural hazards forces coalescing and generating greater destructive forces inflicts imperatives for a comprehensive, integrated risk management framework. In Zimbabwe, disaster management is addressed and coordinated by the disaster management arm, the Civil Protection Unity, which falls under the Ministry of Local Government, Public Works and National Housing. The Ministry of Environment, Water and Climate is also the apex body that works with the Civil Protection Unit to address policy issues and lay guidelines as mandated from the *Civil Protection Act* Chapter 10.06 of 1989. Its primary role is to coordinate all the stakeholders involved in DRM in Zimbabwe, and its main aim is to protect lives, property and the environment from all dangers (Simba 2018). Climate change is being addressed at various levels by other ministries such as Higher Education, Science and Technology and other non-governmental organisations such as the WWF for Nature Zimbabwe and Zimbabwe Environmental Law Association. There is wide divergence among the aforementioned organisations as they operate in silos with an integrated disaster management plan. Disaster risk management in Zimbabwe is sectoral and remains compartmentalised as the policies of various sectors do not harmoniously speak to each other (Ncube-Phiri, Mucherera & Ncube 2015). The Zimbabwe Environmental Law Association deals with marginalised communities to claim their environmental, social, economic and cultural rights within natural resources and the environmental sector. The organisation also deals with climate change and renewable energy programmes. In contrast, the WWF deals with the following thematic areas: wildlife and protected areas management; forestry and landscape management; wetlands management; and renewable energy solutions which fall within the wildlife, forests, freshwater, and climate and energy. This silo mentality of managing various risks posed by anthropogenic and natural factors creates confusion and negates salient protocols to disaster management and CINDs. Muzamhindo and Mutanana (2021) alluded that Zimbabwe has disaster management policies in place, but their implementation and coordination is fragile due to various stakeholders acting independently from each other.

Therefore, there is an urgent need to bring climate change and disaster stakeholders such as non-governmental organisations (NGOs), government institutions, universities, climate change and disaster management activists on a common platform and develop an integrated risk management framework or model for the challenges of climate change and increasing disasters, which are intrinsically linked through societal vulnerability, research and development (R & D) (Matamanda et al. 2017). An integrated approach would entail better coordination among climate change, disasters and development communities. Furthermore, there is a need for improved theoretical and methodological approaches to comprehend and respond to local manifestations of disasters while concurrently addressing the underlying complex global processes through multi-stakeholder involvement (Frederiksen 2017).

## Theoretical framework

### Change theory: Problem, solution and desired outcomes

This study is grounded in the theory of change. The theory-driven evaluation aimed to move beyond a simplistic input-output notion of evaluation. Instead, it requires that programme designers explicitly state how they expect a programme to work, thereby making their implicit assumptions explicit (Reinholz & Andrews 2020). The theory-driven evaluation aimed to move beyond a simplistic input–output notion of evaluation and instead required that programme designers explicitly state how they expected a programme to work, thereby making their implicit assumptions explicit (Reinholz & Andrews 2020). This allows an evaluator to understand better what is being implemented and why making explicit connections between a given intervention and its outcomes. By making the underlying rationale of an initiative explicit, it can be interrogated, assessed and revised systematically as it is being implemented (Reinholz & Andrews 2020).

Theory of change implies that when institutions make efforts to reduce vulnerability and strengthen resilience, countries can protect lives and assets from widely known risks. Disasters can be managed easily if institutions in Zimbabwe have a clear response plan when disaster strikes. This can be achieved if Zimbabwe’s institutions responsible for disaster management have a disaster management plan that is implementable, followed by a vibrant assessment plan to measure success. At the same time, countries can also control the creation of new risks if they implement the right policies and interventions (Chatiza 2019). As countries create sustainable disaster management policies and intervention strategies, there is a need to build innovative institutions to bring change in the climate and DRM domain. Higher education institutions are better placed to work hand in hand with government arms and other relevant stakeholders in facilitating DRM innovation and response strategies. Universities are famous for knowledge creation and their involvement in DRM strategies and policy formulation strongly impacts communities and among various stakeholders (Mawonde 2020).

## Research methodology

The study used a qualitative, exploratory research design. Hamilton and Finley (2019) defined qualitative research methodology as ‘multi-method in its focus, involving an interpretive, naturalistic approach to its subject matter’. An exploratory design is the most appropriate design to the projects focusing on areas where there is the uncertainty of ignorance and where there is a paucity of research on the subject (Bryman 2012). Desktop research was employed to collect DRM and the role of HEIs in CINDs data. A desk study in compiling data that have already been collected reduces data collection costs (Evetts 2014). However, a challenge with desk research is often on the synthesis of the data that needs to be done in a way that does not produce the same results as the previous research or studies. Instead, the logic is to critically analyse the previous studies, extract useful information, validate sources and ultimately align the results with the study at hand (Frederiksen 2017; Munro 2018). Some of the sources that were used include the Council of Higher Education policy documents on DRM curriculum in universities, government documents on DRM and response and peer-reviewed journals from Zimbabwean universities on DRM.

Content analysis and thematic analysis were employed to analyse data gathered from various documents related to Zimbabwe DRM. Bryman (2012:290) defined content analysis as ‘an approach to the analysis of documents and texts that seeks to quantify content in terms of predetermined categories and in a systematic and replicable manner’. Thematic analysis refers to a qualitative research method that can be widely used across a range of epistemologies and research questions (Nowell et al. 2017) for identifying, analysing, organising, describing and reporting themes found within a data set so that the research can produce meaningful findings (Braun & Clarke 2006). The rationale for using both content and thematic analysis is to uncover all the meanings and interpretations of higher education’s contributions in promoting SDGs concerning climate and disaster management. Data analysed was from the World Bank, United Nations reports, national and local newspapers, books and journal articles.

## Results and discussion

The increasing intricacies of climate-related disasters and risks challenge HEIs in Zimbabwe to make pre- and post-disaster efforts by imparting disaster management knowledge to communities. This is a huge task taking into account economic challenges in Zimbabwe due to a myriad and a plethora of factors such as economic sanctions and economic meltdown. These challenges are making it difficult for the government to build strong institutions to deal with disaster management. The meteorological department is one arm of the government, which broadcast weather forecasts and predict extreme weather events with the potential to cause disasters such as floods or droughts. Universities in Zimbabwe have the intellectual capacity to carry out disaster risk-related research culminating in sustainable recommendations. However, universities still face challenges in executing their roles to promote SDGs through DRM. The barriers to achieving SDG goal number 13 (take urgent action to combat climate change and its impact) include insufficient funding and access to the latest technology such as geographic information system (GIS) hardware and software to track and manage disasters effectively. Although the government meteorological department issues early warning information to affected communities, there is a lack of infrastructure, vehicles, helicopters, boats, communication networks in remote areas and strong disaster management teams to evacuate people when disaster strikes, especially during floods. Ultimately, universities are coming up with DRM recommendations, but responsible government institutions lack implementation because of poor funding, corruption and economic sanctions.

### Higher education and sustainable development

The increasing engagement of HEIs in Zimbabwe in the global disaster risk domain reflects a growing commitment to SD. Universities have included sustainability issues in their modules, such as climate change, disaster risk reduction and poverty reduction. Chinhoyi University of Technology (CUT), for example, offers modules with components of climate change significantly in the School of Agricultural Sciences and Technology (SAST) and the School of Wildlife Ecology and Conservation (SWEC) (Kupika et al. 2020). Chinhoyi University of Technology offers a Bachelor of Science Honours Degree in Environmental Science and Technology with modules covering occupational health and safety, sanitation and disaster management skills (CUT 2019). Table 1 indicates universities that have DRM programmes in Zimbabwe.

**TABLE 1:** Disaster risk management curriculum.

Institution	Department	Course level	Module information
BUSE	Geography	BSc. Development Studies BSc. Disaster Management Studies	(DG 410) Disaster Risk Reduction (DG 411) Disaster and Development (DDG 402) Disaster Risk Reduction and Development (DDG 206) Community Based Disaster Risk Reduction
National University of Science Technology (NUST)	Institute of Development Studies	MSc. Disaster Management	(MDM 5101) Hazards, Disasters & the Zimbabwe Emergency Management System (MDM 5102) Disaster Vulnerability and Risk Management (MDM 5302) Emergency Planning, Exercise Design & Evaluation
University of Zimbabwe	Peace Security and Society	MA Disaster Risk Reduction Management Systems	(MDM 503) Disaster Governance (MDM507) Monitoring and Evaluation in Disaster

BUSE, Bindura University of Science Education; DG, development geography; DDG, disaster development geography; MDM, master disaster management.

The Faculty of Arts, Department of Development Studies at Midlands State University (MSU) in Gweru, carried out a project titled: *Climate Change, Disaster Management Institution for Transformation in Zimbabwe, Southern and sub-Saharan Africa Local Rural and Urban Communities,* code named (CC-DM001/20) (MSU 2020). The project themes include disaster management conventions and community development, disaster management institutions and protocols for transformation, theory of disaster management, challenges, humanitarian action and sustainability. The project was meant to tackle disasters associated with climate change and educate wider communities on responding to such catastrophes. In that regard, it is evident that those HEIs see education as a tool for SD and have embraced SD in all their activities. On that note, the teaching and learning, higher education’s contribution can go the extra mile towards building sustainable societies and emanate from engagement with communities, research outputs and the development of green technology that can ensure a balanced type of development (Wu & Shen 2016).

In their study, Shen and Jim Wu (2016) had supported this perspective of higher education for SD. The researchers observed that research trends had indicated the need to integrate higher education teaching and learning, research, curriculum and community engagement towards SD. Trencher, Yarime and Kharrazi (2013) observed that SD is becoming a noble concept and institutions of higher learning are acting as agents in promoting principles of SD in communities.

However, to accelerate the pace to achieve this, HEIs in Zimbabwe and Africa need to be proactive and serve communities with scientific knowledge. The HEIs should not only link theory with practice but they should also allow for potential transformations, act as watchdogs of society and be responsible to the broader community beyond formal education (Trencher et al. 2013). The role of HEIs in SD is so critical that the United Nations designated the decade between 2005 and 2014 as the United Nations Decade of Education for SD (Borys 2010; Togo & Lotz-Sisitka 2013). Although the decade of education for sustainable development (DESD) lapsed, it had raised several universities’ awareness to embrace SD approaches. Moreover, soon after the DESD, the Transform Agenda 2030 kicked in with 17 various SDGs and 169 targets (Mawonde & Togo 2021) to further improve and enhance quality education in societies for SD using SDG 4 (quality education for all).

### Innovation and ICTs by the universities

It is quite essential to appreciate that science, technology and innovation (STI) policy systems have emerged as interconnections between knowledge, values, national and international socio-economic, environmental, technological and organisational components. Universities such as Harare Institute of Technology (HIT), National University Science and Technology (NUST), CUT and Bindura University Science Education (BUSE) are the only institutions with a central bias towards technology courses and programmes in the country, and they have made efforts to create technological tools but little to assist in disaster management. Regardless of that, in 2019, the Government of Zimbabwe launched several innovation hubs at the University of Zimbabwe (UZ), MSU and NUST that seek to transform academic knowledge into adoptable products through research and development. Construction is underway at other universities such as the Great Zimbabwe University (GZU), Zimbabwe National Defence University (ZNDU) and CUT. Moreover, Zimbabwean HEIs have no databases and websites to provide real-time information and data for climate disaster risk reduction (Chirisa & Matamanda 2022).

It is worth to note that the use of Global Positioning Systems (GPS) has not yet been adopted for DRM by universities. The Department of Geography and Environmental Science (DGES) at the UZ is the current leading in geospatial technologies and is in its course and Education 5.0 for full-fledged adoption (Togo & Gandidzanwa 2021). The DGES, for example, used GIS, remote sensing and hydrological modelling techniques to map and model flood-prone areas in Zimbabwe for flood assessment.

Purba (2015) supported adopting information computer systems in universities to predict natural disasters and avert their impact through timeous sustainable planning to minimise economic losses and preserve human lives. The author argues that information communication technology (ICT) is the best choice to overcome obstacles in running HEIs. Baragheh, Rowley and Sambrook (2009) also posited that innovation varies with time and that higher education and learning institutions must use innovation as a tool to respond to climate change and its related threats in the form of extreme weather events. These extreme weather events are responsible for CINDs that threaten humanity and vulnerable countries’ economies. There is a need to increase funding to enable higher education and learning institutions to support activities envisaged under the disaster management pillar and be centres of research and development of new technologies (Tirivangasi & Nyahunda 2019). This study found that universities in Zimbabwe have fewer research programmes. Therefore, there is a need to effectively address climate and disaster management through diverse and integrated research programmes.

### Higher tertiary institutions as sources of information

Historically, disaster risk-related education programmes have dominated in developed countries such as Japan, but the study shows that disaster risk programmes are relatively new and are being taught from undergraduate to Master’s level in Zimbabwe. This study shows that only a handful of HEIs are contributing to generating educational knowledge and technical skills essential for the management of climate adaptation and mitigation in Zimbabwe. The BUSE introduced BSc Honours in Disaster Management, allowing students to undertake research in areas such as Muzarabani, for example, (which is prone to floods) and increase the resilience of local communities to the prevailing hazards through education. The NUST offers a Master of Science in Disaster Management. The programme is critical in building sustainable communities in Zimbabwe. The degree offers the following course modules: MDM 5101 Hazards, Disasters and the Zimbabwe Emergency Management System, MDM 5102 Disaster Vulnerability and Risk Management, MDM 5103 Disaster Education, MDM 5104 Media Management and Disaster Risk Communication, MDM 5201 Earth Sciences and Natural Disasters, MDM 5202 Technological Disasters, MDM 5301 Geographical Information Systems for DRM, MDM 5302 Emergency Planning, Exercise Design and Evaluation, MDM 5303 Public Health Issues in Disaster Management, MDM 5304 Disaster Risk and Emergency Management Regulation (NUST 2020). All these modules are interlinked and they address disaster management issues and challenges following guidelines of the Sendai Framework in responding to disasters and mitigation. Finally, the UZ offers BSc Honours Risk Reduction and Disaster Management. The programme offers modules that respond to various disasters that affect humanity ranging from floods to droughts. The programme addresses humanitarian aid, resilient infrastructure, disaster communication networks, disaster monitoring and control and evaluation.

It is worth noting that BUSE introduced a Master of Science in Climate Change and SD, a programme that incorporates climate change issues. The Catholic University in Zimbabwe also offers a short course on disaster management. Such a move by the institution builds a much stronger emphasis and the integration of climate change and disaster management agenda into all aspects of university activities for the institution to be relevant and respond to prevailing challenges. Higher educational institutions have played a crucial role in the climate change and disaster risk domain.

### Public awareness

The HEIs in Zimbabwe are generating knowledge about disaster risks, the factors that lead to disasters and the actions that can be taken to reduce exposure to flooding, drowning and lightning hazards (Mutsau & Billiat 2015). Local universities (e.g. BUSE, NUST, UZ, MSU) have developed public awareness for disaster risk reduction through community engagement in rural and urban areas, enhancing community preparedness. The HEIs in Zimbabwe have organised conferences where leading academics, scientists and researchers exchange and share their experiences, knowledge and research results, which is equally important to promote SD. In 2016, NUST hosted the Third Biennial Conference of the Southern Africa Society for Disaster Risk Reduction. Climate change and disaster awareness are helpful in capacity building and are necessary for Zimbabwe to fulfil her commitment under the United Nations Framework Convention on Climate Change (Brown et al. 2012).

Some communities (especially in Muzarabani Mashonaland Central Province in Zimbabwe) have been exposed to floods and droughts over the years (Mucherera & Mavhura 2020), and it is important to develop coping mechanisms and strategies. Hence, university awareness programmes empower communities and enable indigenous knowledge to be incorporated in climate change and disaster management. However, universities in Zimbabwe are challenged to implement more disaster risk management programmes in a cross-disciplinary manner. Therefore, the introduction of new programmes should incorporate elements of interdisciplinary and transdisciplinary.

According to Mashizha, Monga and Dzvimbo (2017), education and awareness about CINDs and sustainable coping mechanisms were meagre among most smallholder farmers partly due to weak institutional coordination and support. Peers’ climate change education and values create a prerogative and positive influence on neighbouring rural people to adopt sustainable mitigatory strategies, which appear successful from other climate knowledgeable farmers or stakeholders (Mashizha et al. 2017). Perceptions and understanding of climate change disasters greatly influence awareness and disaster management involvement in the sustainable interventions being executed by various stakeholders.

Mullens (2018) and Dzvimbo et al. (2017) observed that several hazards derail available climate and weather information from penetrating rural communities, including scientific jargon, inadequate dissemination channels and poorly formalised institutional frameworks at the national level. These barriers or hazards have created gaps between scientists, researchers, policymakers and communities, yet all these stakeholders should cooperate to ensure that climate change and its impacts are understood and mitigated. In this regard, one of the critical roles of higher learning institutions is to simplify scientific information or knowledge to the public.

More so, HEIs have not crafted emergency plans for poor and vulnerable community preparedness in Zimbabwe except for awareness on floods, evacuation, search and rescues. The HEIs must embrace both scientific and indigenous knowledge as community-based approaches to disaster management and focus much on rural areas where there is inadequate information about the dynamics of climate disasters (Baumwoll 2008).

### Scientific research and advisory services

The UZ provides technical assistance for scientific research to the government and or policymakers (Nyambi & Maynard 2012). The UZ has been involved in studying soil sampled in communities that are vulnerable to flooding and developed sustainable construction materials. There is also a steady increase in undergraduate students in research areas but Doctoral and Master’s students still dominate. Although different factors drove scholars and researchers to be involved in SD research initiatives, a lot still needs to be performed. There is limited research in centres of Zimbabwe HEIs to assist in disaster risk reduction (Mutsau & Billiat 2015). The role of the Centre of Applied Social Sciences (CASS) at the UZ in socio-ecological studies and research of wetland conservation is increasing and becoming their area of research focus to control flooding as wetlands are known as water infiltration sites.

The UZ introduced the biannual *Journal of Urban Systems and Innovation for Resilience* in 2019 as a platform for researchers to air practical issues in Zimbabwe’s urban communities such as climate change. This journal platform produces local climate change and disaster risk and management from lived experiences of various communities such as those in the Lowveld. These communities include Chiredzi, Muzarabani, Guruve district and the Zambezi basin. These areas are often affected by severe flooding and research information is helpful to other stakeholders facing similar challenges. Research institutions in Zimbabwe are mainly dominated by formal education and training, although informal occurs through field trips and visits to sites.

Empirical research funding has been cited as one of the most impediments to undertaking reliable researches by university scholars (Mawonde & Togo 2021). Despite climate change education, the study found that limited financial resources are usually allocated to climate change-related research within HEIs (Mawonde & Togo 2019). Sustainable development research is critical to ensure that climate change solutions are far-reaching to affected communities to improve their living standards. Universities various colleges and departments need to be financially strengthened to increase vibrant institutional climate change research to avert negative impacts of extreme weather events. Civil Protection Authority monopoly in managing government disaster funds leads to failure to grapple with disasters, and HEIs must be incorporated with their superior knowledge and varied expertise to bolster DRM and reduction (Rwodzi 2019).

### Higher learning organisations and networks: A liaison question

Institutions of higher learning are increasingly getting into a relationship with research stakeholders, such as the Research Council of Zimbabwe and the Scientific Institute of Research and Development Cooperation. However, evidence shows that currently, there are no solid and extensive networks of institutions to drive the common cause of disaster management and risk reduction. Networks are essential in countering setbacks and facilitating disaster risk identification, mapping and communication about the risks (Owens 2017). For higher education to play a revitalised role in the SD framework, there is a great need for publicly funded research and higher education partnerships (Owens 2017). Understanding climate disaster risks as a body of academics, policymakers, practitioners, and the public goes a long way in finding lasting solutions to minimise the extent of damage and losses posed by this catastrophic phenomenon.

### Scaling up resilience through tertiary institutions

Universities play a critical role in scaling up the resilience of vulnerable communities to disasters and natural hazards (Amri et al. 2017). In particular, the study observed that besides passing the knowledge and training students to become disaster risk practitioners, HEIs in Zimbabwe are also training communities on disaster concepts, disaster prevention, mitigation, preparedness and response (Mavhura et al. 2013).

Bindura University of Science Education has shown its commitment to building people’s resilience in Chadereka village in Muzarabani. The area experienced catastrophic floods in 2000 and 2007, which presented several vital lessons. The university has mastered how to respond to emergencies and how local communities must have the necessary disaster education and skills to be in positions of readiness in the event of a new disaster. Resilience building is a bottom-up approach in which communities at the grassroots need to ensure that we build effective response mechanisms (Odemerho 2015). This implies that universities can play an essential part in reducing the negative impacts of extreme climate events in direct and indirect ways (Abedin & Shaw 2015).

With the world’s increasing frequency and intensity of disasters, the need for painstakingly concerted efforts towards building disaster-resilient communities in Zimbabwe cannot be ignored. Thus, higher education’s responsibility is to build competencies for SD among vulnerable communities. The role of universities should not end at building community resilience but go beyond empowering communities to become leaders for SD (Abedin & Shaw 2015). Such empowerment can be demonstrated through capacity building. Among all the recommended actions universities should embrace towards SD, such as research and education (Adams, Martin & Boom 2018; Lozano et al. 2015), capacity building has gained much research attention (Hooge & Van Dam 2019).

Recent drought and flood warnings by the Meteorological Services Department (MSD) have highlighted the need for an intensification of initiatives to address loss and damage and the entire scope of disaster reduction responsiveness. Therefore, as HEIs are important community stakeholders, there are building communities’ preparedness to handle environmental disaster situations that have been slow and inadequate, mainly because of poor funding. In low-lying areas such as Muzarabani, Tsholotsho, Chisumbanje and Chibuwe, and other areas of Matebeleland North such as Hwange and Binga Districts and Eastern Highlands of Zimbabwe such as Chimanimani District, flooding is prone and households are at risk of hunger (Mtapuri, Dube & Matunhu 2018). The Southern parts of the country are prone to droughts in successive years.

From 2016 to 2019, low-lying and other areas of the country have been vulnerable to floods. Recently, Chimanimani, Chipinge and some areas of Masvingo were hit by the recent cyclones and Hwange District was affected by flash floods. Tsholotsho experienced devastating flash floods and the government was not able to cope with the catastrophe (Mtapuri et al. 2018). In that regard, HEIs must spell out strategies to deal with such catastrophes. Currently, Zimbabwe and most of its HEIs are struggling to tackle disaster management as a discipline due to lack of funding, among other challenges.

### Student activism for disaster action

One of the areas where HEIs are lacking is active student engagement and activism in climate disaster management to address the loopholes at the HEIs and relevant state and non-state departments. Students are vibrant and energetic change agents (Mawonde & Togo 2021) with high energy levels to implement change as future environmental leaders (Mcmillin & Dyball 2009). It is worth stressing that student activism is critical to bringing the quality of education and programmes to HEIs and relevant authorities to account for their deeds and misdeeds in handling disasters across the country. For instance, in 2019, in response to the Cyclone Idai disaster, no single HEIs student rose to action to push the universities, national and local governments to curtail the disaster, including alerting the people and restoring livelihoods (Zhanda, Dzvimbo & Chitongo 2021). Therefore, there is a need of not solely confining students’ activism to power politics.

## Conclusion and the way forward

This article found that HEIs are essential climate and disaster management partners in Zimbabwe. Tertiary education institutions act as magnets that build adaptive community capacity to climate change. Knowledge and education turn out to be more important than income in reducing disaster vulnerability.

It can also be concluded that universities can integrate and align their efforts with government policy towards disaster management and mitigations. To date, HEIs in Zimbabwe have done an initial flurry on disaster management. Through their research, HEIs and scholars from those institutions directly influence risk perceptions, skills and knowledge. The HEIs in Zimbabwe should build their disaster satellite tracking infrastructure to create domestic technology solutions to local development challenges and support technology transfer, adaptation and dissemination for SD and resilience. For HEIs in Zimbabwe to inculcate sound and relevant environmental disaster knowledge, quality of institutions, graduate experiences and more research outputs are primarily required. Based on the empirical evidence from this study, it is interesting to observe that university-community partnerships can quicken the pace and strengthen community resilience. Higher education institutions’ core mission includes public engagement to advance achieving SDGs.

### Recommendations

Henceforth, this article recommends that climate and disaster management policies focus more on the general empowerment of communities through education, human capital formation and capacity building as the most efficient strategy towards building resilience. This is commendable, and HEIs, through their curriculum, teaching and learning, research and community engagement, can use students as agents of change to conscientise communities on the need to understand climate change and CINDs and ways to minimise them.

Through research publications inclined to wetlands and flooding, HEIs should be supported with incentives to lure scholars to come up with cross-cutting research that will significantly improve the preservation of wetlands to control flooding, which often affects Zimbabwe’s low lying areas. Hence, it is worthy of integrating environment and development into education activities from primary school to tertiary institutions of which appropriate methods and materials will be developed to respond to extreme weather events and disasters.

This should be performed to establish national centres for excellence and interdisciplinary research and education in environment and development (Chigundu 2018). Besides, HEIs should consider enrolment to programmes such as Accounting and Finance with courses in disaster financing, auditing, levying and monitoring. The HEIs are supposed to set up interdisciplinary research units and organisations to address prevailing climate and disaster management challenges.

The awareness and knowledge generated by HEIs need to be reflected in sustainability science and HEIs must collaborate with communities in capacity building to enable transformation. Public awareness of climate change is an essential measure for influencing the whole society to jointly participate in activities to mitigate and adapt to climate change (Demuzere et al. 2014).

All the relevant departments and colleges of higher education and learning need to contribute significantly towards inventing tools to deal with climate change disasters, such as early warning systems. More so, local universities should be in the front line to advise and urge local authorities and other government departments, such as the Civil Protection Department, to use technology for disaster risk preparedness and response (Chirisa et al. 2016).

Students must be encouraged and given support (financially or in other ways) to undertake training in SD and support their research through incentives such as research grants. This enhances improved research outputs in SD. Many positive outcomes can be realised if climate change and disaster management are given maximum support in Zimbabwe.

As the education policy shift, Zimbabwe is shunning Education 3.0, which focuses on community service, research, and teaching in favour of Education 5.0, which broadens the space and innovation hubs that focus on sustainability thematic study areas by the universities. There is a need to seize the opportunity and embrace many climate-related disaster concepts for SD.

## References

[CIT0001] Abedin, M.A. & Shaw, R., 2015, ‘The role of university networks in disaster risk reduction: Perspective from coastal Bangladesh’, *International Journal of Disaster Risk Reduction* 13, 381–389. 10.1016/j.ijdrr.2015.08.001

[CIT0002] Adams, R., Martin, S. & Boom, K., 2018, ‘University culture and sustainability: Designing and implementing an enabling framework’, *Journal of Cleaner Production* 171, 434–445. 10.1016/j.jclepro.2017.10.032

[CIT0003] Africa Union Commission, 2015, *Agenda 2063: The Africa we want*, African Union, Addis Ababa.

[CIT0004] Aitsi-Selmi, A., Egawa, S., Sasaki, H., Wannous, C. & Murray, V., 2015, ‘The Sendai framework for disaster risk reduction: Renewing the global commitment to people’s resilience, health, and well-being’, *International Journal of Disaster Risk Science* 6(2), 164–176. 10.1007/s13753-015-0050-9

[CIT0005] Albareda-Tiana, S., Ruíz-Morales, J., Azcárate, P., Valderrama-Hernández, R. & Múñoz, J.M., 2020, ‘The EDINSOST project: Implementing the sustainable development goals at university level’, in *Universities as living labs for sustainable development*, pp. 193–210, Springer, Cham.

[CIT0006] Amri, A., Bird, D.K., Ronan, K., Haynes, K. & Towers, B., 2017, ‘Disaster risk reduction education in Indonesia: Challenges and recommendations for scaling up’, *Natural Hazards and Earth System Sciences* 17(4), 595–612. 10.5194/nhess-17-595-2017

[CIT0007] Baragheh, A., Rowley J. & Sambrook, S., 2009, ‘Towards a multidisciplinary definition of innovation’, *Management Decision* 47(8), 1323–1339. 10.1108/00251740910984578

[CIT0008] Baumwoll, J., 2008, *The value of indigenous knowledge for disaster risk reduction: A unique assessment tool for reducing community vulnerability to natural disasters*, Webster University, Webster Groves, MO.

[CIT0009] Benson, C., 2016, *Promoting sustainable development through disaster risk management*, Asian Development Bank, Mandaluyong.

[CIT0010] Bhowmik, J., Selim, S.A. & Huq, S., 2017, *The role of Universities in achieving the sustainable development goals*, CSD-ULAB and ICCCAD Policy Brief, ULAB, Dhaka.

[CIT0011] Blewitt, J., 2012, *Understanding sustainable development*, Routledge, London.

[CIT0012] Borys, T., 2010, ‘Decade of education for sustainable development – Polish challenges’, *Problems of Sustainable Development* 5(1), 59–70.

[CIT0013] Braun, V. & Clarke, V., 2006, ‘Using thematic analysis in psychology’, *Qualitative Research in Psychology* 3, 77–101. 10.1191/1478088706qp063oa

[CIT0014] Brennan, J., King, R. & Lebeau, Y., 2004, ‘The role of universities in the transformation of societies’, Synthesis report, Centre for Higher Education Research and Information/Association of Commonwealth Universities, London.

[CIT0015] Brown, D., Chanakira, R.R., Chatiza, K., Dhliwayo, M., Dodman, D., Masiiwa, M. et al., 2012, *Climate change impacts, vulnerability and adaptation in Zimbabwe*, International Institute for Environment and Development, London.

[CIT0016] Bryman, A., 2012, *Social science research methods*, 4th edn., Oxford University Press, Oxford.

[CIT0017] Chatiza, K., 2019, *Cyclone Idai in Zimbabwe: An analysis of policy implications for post-disaster institutional development to strengthen disaster risk management*, Oxfam, Oxford.

[CIT0018] Chigundu, A., 2018, ‘Removal of coloured organic pollutants from wastewaters by magnetite/carbon nanocomposites: Single and binary systems’, *Journal of Chemistry* 2018, 6249821. 10.1155/2018/6249821

[CIT0019] Chirisa, I., Bandauko, E., Mazhindu, E., Kwangwama, N.A. & Chikowore, G., 2016, ‘Building resilient infrastructure in the face of climate change in African cities: Scope, potentiality and challenges’, *Development Southern Africa* 33(1), 113–127. 10.1080/0376835X.2015.1113122

[CIT0020] Chirisa, I. & Dumba, S., 2012, ‘Spatial planning, legislation and the historical and contemporary challenges in Zimbabwe: A conjectural approach’, *Journal of African Studies and Development* 4(1), 1–13.

[CIT0021] Chirisa, I. & Matamanda, A.R., 2022, ‘Science communication for climate change disaster risk management and environmental education in Africa’, in *Research anthology on environmental and societal impacts of climate change*, pp. 636–652, IGI Global, Hershey, PA.

[CIT0022] Connell, J.P. & Kubisch, A.C., 1998, ‘Applying a theory of change approach to the evaluation of comprehensive community initiatives: Progress, prospects, and problems’, *New Approaches to Evaluating Community Initiatives* 2(15–44), 1–16.

[CIT0023] Crompton, R. & McAneney, J., 2008, ‘The cost of natural disasters in Australia: The case for disaster risk reduction’, *Australian Journal of Emergency Management* 23(4), 43–46.

[CIT0024] Cui, Y., 2016, *Genetically modified rodent models and environmental management*, Routledge, Seattle, DC.

[CIT0025] CUT, 2019, *Chinhoyi University of Technology Bachelor of Science Honours Degree in Environmental Science and Technology*, viewed from 12 December 2021, from https://zwadmissions.com/chinhoyi-university-of-technology-bachelor-of-science-honours-degree-in-environmental-science-and-technology/.

[CIT0026] De La Poza, E., Merello, P., Barberá, A. & Celani, A., 2021, ‘Universities’ reporting on SDGs: Using the impact rankings to model and measure their contribution to sustainability’, *Sustainability* 13(4), 2038. 10.3390/su13042038

[CIT0027] Demuzere, M., Orru, K., Heidrich, O., Olazabal, E., Geneletti, D., Orru, H. et al., 2014, ‘Mitigating and adapting to climate change: Multi-functional and multi-scale assessment of green urban infrastructure’, *Journal of Evironmental Management* 146, 107–115. 10.1016/j.jenvman.2014.07.025 25163601

[CIT0028] Diakakis, M., Boufidis, N., Grau, J.M.S., Andreadakis, E. & Stamos, I., 2020, ‘A systematic assessment of the effects of extreme flash floods on transportation infrastructure and circulation: The example of the 2017 Mandra flood’, *International Journal of Disaster Risk Reduction* 47, 101542. 10.1016/j.ijdrr.2020.101542

[CIT0029] Dzvimbo, M.A., Mashizha, T.M., Monga, M. & Ncube, C., 2017, ‘Conservation agriculture and climate change: Implications for sustainable rural development in Sanyati, Zimbabwe’, *Journal of Social and Development Sciences* 8(2), 38–46. 10.22610/jsds.v8i2.1795

[CIT0030] Evetts, J., 2014, ‘The concept of professionalism: Professional work, Professional practice and learning’, in S. Billett, C. Harteis & H. Gruber (eds.), *International handbook of research in professional and practice-based learning*, pp. 29–56, Springer, New York, NY.

[CIT0031] Fitzgerald, G., Rego, J., Ingham, V., Brooks, B., Cottrell, A., Manock, I. et al., 2017, ‘Teaching emergency and disaster management in Australia: Standards for higher education providers’, *The Australian Journal of Emergency Management* 32(3), 22–23. 10.1017/S1049023X17002552

[CIT0032] Frederiksen, M., 2017, *Delivering better development: The role of the urban and rural planner*, Global Planners Network, London.

[CIT0033] Garwe, E.C. & Thondhlana, J., 2019, ‘Higher education systems and institutions, Zimbabwe’, in J.C. Shin & P. Teixeira (eds.), *Encyclopedia of international higher education systems and institutions*, pp. 2–4, Springer, Dordrecht.

[CIT0034] Graham, C., 2020, ‘Climate-induced population displacement in sub-Saharan Africa: A review of resilience-building strategies’, *Geoforum* 117, 300–303. 10.1016/j.geoforum.2020.07.004

[CIT0035] Hamilton, A.B. & Finley, E.P., 2019, ‘Qualitative methods in implementation research: An introduction’, *Psychiatry Research* 280, 112516. 10.1016/j.psychres.2019.112516 31437661 PMC7023962

[CIT0036] Hooge, I.E & Van Dam, Y.K., 2019, ‘Reach out and touch: Student training community projects for sustainability – A case study’, *Sustainability in Higher Education* 20(7), 1278–1289. 10.1108/IJSHE-11-2018-0222

[CIT0037] Jomo, K.S., Chowdhury, A., Sharma, K. & Platz, D., 2016, *Public-private partnerships and the 2030 Agenda for Sustainable Development: fit for purpose?*, DESA Working Paper No. 148, New York, NY.

[CIT0038] King, E., 2011, *Education is fundamental to development and growth*, The World Bank, Washington, DC.

[CIT0039] Kupika, O.L., Mbereko, A. & Chinokwetu, V., 2020, ‘Role of universities towards achieving climate change-related SDGs: Case of Chinhoyi University of Technology, Zimbabwe’, in G. Nhamo & V. Mjimba (eds.), *Sustainable development goals and institutions of higher education*, pp. 97–110, Sustainable Development Goals Series, Springer, Cham.

[CIT0040] Kwauk, C., 2020, ‘Roadblocks to quality education in a time of climate change’, Brief, Center for Universal Education at the Brookings Institution.

[CIT0041] Leal Filho, W., Morgan, E.A., Godoy, E.S., Azeiteiro, U.M., Bacelar-Nicolau, P., Ávila, L.V. et al., 2018, ‘Implementing climate change research at universities: Barriers, potential and actions’, *Journal of Cleaner Production* 170, 269–277. 10.1016/j.jclepro.2017.09.105

[CIT0042] Lee, J., 2020, ‘The human right to education: Definition, research and annotated bibliography’, *Emory International Law Review* 34, 757.

[CIT0043] Lozano, R., Ceulemans, K., Alonso-Almeida, M., Huisingh, D., Lozano, F.J., Waas, T. et al., 2015, ‘A review of commitment and implementation of sustainable development in higher education: Results from a worldwide survey’, *Journal of Cleaner Production* 108, 1–18. 10.1016/j.jclepro.2014.09.048

[CIT0044] Mashizha, T.M., Monga, M. & Dzvimbo, M.A., 2017, ‘Improving livelihoods of resettled farmers through development of a knowledge base on climate change in Mhondoro-Ngezi District, Zimbabwe’, *International Journal of Sustainable Development Research* 3(2), 18–26. 10.11648/j.ijsdr.20170302.12

[CIT0045] Matamanda, A.R., Dzvimbo, M.A. & Kadebu, R.T., 2017, ‘Climate change and infrastructure planning: Implications for sustainable urban management in Harare, Zimbabwe’, *Journal of Public Administration and Development Alternatives* 2(1), 95–111.

[CIT0046] Mavhura, E., Manyena, S.B., Collins, A.E. & Manatsa, D., 2013, ‘Indigenous knowledge, coping strategies and resilience to floods in Muzarabani, Zimbabwe’, *International Journal of Disaster Risk Reduction* 5, 38–48. 10.1016/j.ijdrr.2013.07.001

[CIT0047] Mawonde, A., 2020, ‘Involvement of students in campus greening initiatives: The case of the University of South Africa’, Unpublished thesis.

[CIT0048] Mawonde, A. & Togo, M., 2019, ‘Implementation of SDGs at the University of South Africa’, *International Journal of Sustainability in Higher Education* 20(5), 932–950. 10.1108/IJSHE-04-2019-0156

[CIT0049] Mawonde, A. & Togo, M., 2021, ‘Challenges of involving students in campus SDGs-related practices in an ODeL context: The case of the University of South Africa (Unisa)’, *International Journal of Sustainability in Higher Education* 22(7), 1487–1502. 10.1108/IJSHE-05-2020-0160

[CIT0050] McMillin, J. & Dyball, R., 2009, ‘Developing a whole-of-university approach to educating for sustainability: Linking curriculum, research and sustainable campus operations’, *Journal of Education for Sustainable Development* 3(1), 55–64.

[CIT0051] Mensah, J., 2019, ‘Sustainable development: Meaning, history, principles, pillars, and implications for human action: Literature review’, *Cogent Social Sciences* 5(1), 1653531. 10.1080/23311886.2019.1653531

[CIT0052] Messerli, P., Kim, E.M., Lutz, W., Moatti, J.P., Richardson, K., Saidam, M. et al., 2019, ‘Expansion of sustainability science needed for the SDGs’, *Nature Sustainability* 2(10), 892–894. 10.1038/s41893-019-0394-z

[CIT0053] MSU, 2020, *Climate change, disaster management institution for transformation in Zimbabwe, Southern and sub-Saharan*, viewed 12 December 2021, from https://ww5.msu.ac.zw/download/climate-change-disaster-management-institution-for-transformation-in-zimbabwe-southern-and-sub-saharan-africa-local-rural-and-urban-communities/.

[CIT0054] Mtapuri, O., Dube, E. & Matunhu, J., 2018, ‘Flooding and poverty: Two interrelated social problems impacting rural development in Tsholotsho district of Matabeleland North province in Zimbabwe’, *Jàmbá: Journal of Disaster Risk Studies* 10(1), 1–7. 10.4102/jamba.v10i1.455 PMC6014082 29955259

[CIT0055] Mucherera, B. & Mavhura, E., 2020, ‘“Flood survivors” perspectives on vulnerability reduction to floods in Mbire district, Zimbabwe’, *Jàmbá: Journal of Disaster Risk Studies* 12(1), a663. 10.4102/jamba.v12i1.663 PMC7136695 32284813

[CIT0056] Mullens, A., 2018, *Youth wetlands education and outreach program*, viewed 24 June 2018, from http://urban4hscience.rutgers.edu/sustained-science-programs/wetlands.html.

[CIT0057] Munro, A., 2018, *Superpower*, Encyclopaedia Britannica.

[CIT0058] Mutsau, S. & Billiat, E., 2015, ‘Leveraging schools systems as a locus for disaster risk reduction in Zimbabwe’, *Journal of Education and Practice* 6(29), 163–169.

[CIT0059] Muzamhindo, T.E. & Mutanana, N., 2021, ‘Child-sensitivity mechanisms in disaster risk management interventions in Zimbabwe’s Cyclone Idai prone areas’, *International Journal of Research in Humanities and Social Studies* 8(7), 17–29.

[CIT0060] Ncube-Phiri, S., Mucherera, B. & Ncube, A., 2015, ‘Artisanal small-scale mining: Potential ecological disaster in Mzingwane District, Zimbabwe’, *Jàmbá: Journal of Disaster Risk Studies* 7(1), 1–11. 10.4102/jamba.v7i1.158 PMC6014108 29955279

[CIT0061] Nhede, N.T., 2013, ‘The Zimbabwe growth point phenomenon: Impact and implications on public service delivery’, *Administration Publica* 21(4), 117–129.

[CIT0062] Nowell, L.S., Norris, J.M., White, D.E. & Moules, N.J., 2017, ‘Thematic analysis: Striving to meet the trustworthiness criteria’, *International Journal of Qualitative Methods* 16(1). 10.1177/1609406917733847

[CIT0063] NUST, 2020, *Master of Science Degree in disaster management programme*, viewed 12 December 2021, from https://www.nust.ac.zw/index.php/component/content/article/120-postgraduate-degree-programmes/commerce-postgrad/305-master-of-science-degree-in-disaster-management.html?Itemid=437.

[CIT0064] Nyambi, E. & Maynard, S., 2012, ‘An investigation of institutional repositories in state universities in Zimbabwe’, *Information Development* 28(1), 55–67. 10.1177/0266666911425264

[CIT0065] Odemerho, F.O., 2015, ‘Building climate change resilience through bottom-up adaptation to flood risk in Warri, Nigeria’, *Environment and Urbanization* 27(1), 139–160. 10.1177/0956247814558194

[CIT0066] Owens, T.L., 2017, ‘Higher education in the sustainable development goals framework’, *European Journal of Education* 52, 414. 10.1111/ejed.12237

[CIT0067] Pappas, E., 2012, ‘A new systems approach to sustainability: University responsibility for teaching sustainability in contexts’, *Journal of Sustainability Education* 3(1), 3–18.

[CIT0068] Pearson, L. & Pelling, M., 2015, ‘The UN Sendai framework for disaster risk reduction 2015–2030: Negotiation process and prospects for science and practice’, *Journal of Extreme Events* 2(1), 1571001. 10.1142/S2345737615710013

[CIT0069] Purba, T.J., 2015, ‘Applied ICT innovation services of academic information system: Strategy for higher education management’, in *2015 International Conference of Organisational Innovation*, pp. 841–855, ICOI, s.l.

[CIT0070] Reinholz, D.L. & Andrews, T.C., 2020, ‘Change theory and theory of change: What’s the difference anyway?’, *International Journal of STEM Education* 7(1), 1–12. 10.1186/s40594-020-0202-3

[CIT0071] Rwodzi, A., 2019, ‘Chapter eight climatological anomalies, food politics and the poor in Africa’, in *Necroclimatism in a spectral world (dis) order?: Rain petitioning, climate and weather engineering in 21st century Africa*, p. 231, s.n., s.l.

[CIT0072] Shen, J. & Jim Wu, Y., 2016, ‘Higher education for sustainable development: A systematic review’, *International Journal of Sustainability in Higher Education* 17(5), 633–651. 10.1108/IJSHE-01-2015-0004

[CIT0073] Simba, F.M., 2018, ‘Zimbabwe’s preparedness in managing meteorological disasters: A case of applying disaster risk management in managing impacts of climate change’, *Journal of Geography & Natural Disasters* 8, 231. 10.4172/2167-0587.1000231

[CIT0074] Tanesab, J.P., 2020, ‘Institutional effectiveness and inclusions: Public perceptions on Indonesia’s disaster management authorities’, *International Journal of Disaster Management* 3(2), 1–15. 10.24815/ijdm.v3i2.17621

[CIT0075] Tirivangasi, H.M. & Nyahunda, L., 2019, ‘Challenges faced by rural people in mitigating the effects of climate change in the Mazungunye communal lands, Zimbabwe’, *Jàmbá: Journal of Disaster Risk Studies* 11(1), 1–9. 10.4102/jamba.v11i1.596 PMC6407465 30863509

[CIT0076] Togo, M. & Gandidzanwa, C.P., 2021, ‘The role of education 5.0 in accelerating the implementation of SDGs and challenges encountered at the University of Zimbabwe’, *International Journal of Sustainability in Higher Education* 22(7), 1520–1535. 10.1108/IJSHE-05-2020-0158

[CIT0077] Togo, M. & Lotz-Sisitka, H., 2013, ‘The unit-based sustainability assessment tool and its use in the UNEP mainstreaming environment and sustainability in African universities partnership’, in *Sustainability assessment tools in higher education institutions*, pp. 259–288, Springer, Cham.

[CIT0078] Trencher, G.P., Yarime, M. & Kharrazi, A., 2013, ‘Co-creating sustainability: Cross-sector university collaborations for driving sustainable urban transformations’, *Journal of Cleaner Production* 50, 40–55. 10.1016/j.jclepro.2012.11.047

[CIT0079] Trencher, G.P., Yarime, M., McCormick, K.B., Doll, C.N. & Kraines, S.B., 2014, ‘Beyond the third mission: Exploring the emerging university function of co-creation for sustainability’, *Science and Public Policy* 41(2), 151–179. 10.1093/scipol/sct044

[CIT0080] UNISDR Hyogo Framework for Action, 2007, *Hyogo Framework for Action 2005–2015: Building the resilience of nations and communities to disasters*, viewed 03 May 2020, from http://www.unisdr.org/we/coordinate/hfa.

[CIT0081] United Nations Educational, Scientific and Cultural Organisation (UNESCO), 2014, *Sustainable development begins with education: How education can contribute to the proposed post-2015 goals*, UNESCO, Paris.

[CIT0082] United Nation (UN), 2016, *UN’s 2016 year in review: Challenges and milestones for the international community*, UN, New York, NY.

[CIT0083] United Nations Office for Disaster Risk Reduction (UNISDR), 2009, *Terminology on disaster risk reduction*, viewed 01 May 2020, from https://www.undrr.org/publication/2009-unisdr-terminology-disaster-risk-reduction.

[CIT0084] United Nations Office for Disaster Risk Reduction (UNISDR), 2015, ‘Sendai framework for disaster risk reduction 2015–2030’, in *Proceedings of the 3rd United Nations World Conference on DRR*, vol. 1, Sendai, Japan.

[CIT0085] WCED, 1987, *World commission on environment and development (WCED): Our common future*, Oxford University Press, London.

[CIT0086] Wu, Y.C.J. & Shen, J.P., 2016, ‘Higher education for sustainable development: A systematic review’, *International Journal of Sustainability in Higher Education* 17(5), 633–651. 10.1108/IJSHE-01-2015-0004

[CIT0087] Zhanda, K., Dzvimbo, M.A. & Chitongo, L., 2021, ‘Children climate change activism and protests in Africa: Reflections and lessons from Greta Thunberg’, *Bulletin of Science, Technology & Society* 41(4), 87–98.

